# Irradiation-induced brain senescence accelerates cardiac aging via systemic mechanisms: insights from transcriptomic profiling

**DOI:** 10.1007/s11357-025-01953-7

**Published:** 2025-10-26

**Authors:** Rafal Gulej, Roland Patai, Tamas Kiss, Siva Sai Chandragiri, Shoba Ekambaram, Raghavendra Yelahanka Nagaraja, Dorina Nagy, Kiana Vali Kordestan, Tamas Lakat, Stefano Tarantini, Peter Mukli, Anna Ungvari, Andriy Yabluchanskiy, Zoltan Benyo, Anna Csiszar, Zoltan Ungvari

**Affiliations:** 1 Vascular Cognitive Impairment, Neurodegeneration and Healthy Aging Program, Department of Neurosurgery, University of Oklahoma Health Sciences Center, Oklahoma City, OK USA; 2 Oklahoma Center for Geroscience and Healthy Brain Aging, University of Oklahoma Health Sciences Center, Oklahoma City, OK USA; 3 International Training Program in Geroscience, Doctoral College/Institute of Preventive Medicine and Public Health, Semmelweis University, Budapest, Hungary; 4 Pediatric Center, MTA Center of Excellence, Semmelweis University, Budapest, Hungary; 5 Cerebrovascular and Neurocognitive Diseases Research Group, HUN-REN-SU, Semmelweis University, Budapest, 1094 Hungary; 6 Diabetes Research Group, MTA-SE Lendület “Momentum”, Budapest, Hungary; 7 Department of Health Promotion Sciences, College of Public Health, University of Oklahoma Health Sciences Center, Oklahoma City, OK USA; 8 The Peggy and Charles Stephenson Cancer Center, University of Oklahoma Health Sciences Center, Oklahoma City, OK USA; 9 Institute of Preventive Medicine and Public Health, Semmelweis University, Budapest, Hungary; 10 Fodor Center for Prevention and Healthy Aging, Semmelweis University, Budapest, Hungary; 11 International Training Program in Geroscience, Doctoral School of Basic and Translational Medicine/Institute for Translational Medicine, Semmelweis University, Budapest, Hungary

**Keywords:** Brain senescence, Hypothalamus, DNA damage, Systemic aging, Cell non-autonomous aging, Senescence-associated secretory phenotype, SASP, Neuroendocrine, Endocrine, Transcriptomics, Cardiac aging, Neuroinflammation, Circulating factors, Cardiovascular aging

## Abstract

Aging is characterized by a coordinated functional decline across multiple organs. While cell-autonomous mechanisms contribute to local aging phenotypes, the systemic synchronicity of aging suggests a major role for cell non-autonomous drivers. Emerging evidence implicates the hypothalamus—a central regulator of neuroendocrine and homeostatic functions—as a potential source of circulating pro-geronic signals. A hallmark of brain aging is the accumulation of senescent cells, particularly in microglia and brain microvascular endothelial cells, including within the hypothalamus, which contributes to a heightened state of neuroinflammation and altered systemic signaling. Here, we tested the hypothesis that brain senescence and its associated inflammatory milieu promote peripheral aging by reshaping the systemic environment. To model this, we employed targeted whole-brain irradiation (WBI) in young mice—a well-established method to induce widespread brain cellular senescence and neuroinflammation, mimicking changes seen in natural aging. Two months after WBI, we performed transcriptomic profiling of the heart to evaluate remote, cell non-autonomous effects. Cardiac RNA sequencing revealed a striking overlap in gene expression changes between WBI-treated young mice and naturally aged controls. Notably, several gene sets associated with fundamental cellular and molecular mechanisms of aging were concordantly dysregulated in both groups, with strong enrichment for pathways related to mitochondrial metabolism, immune activation, interferon signaling, and extracellular matrix remodeling. These findings demonstrate that localized brain senescence is sufficient to induce aging-like transcriptomic remodeling in peripheral organs, likely mediated by circulating factors. Our findings establish brain senescence as a key orchestrator of systemic aging—a mechanism that may contribute to accelerated aging trajectories in individuals with lifestyle-associated increased brain senescence and neuroinflammation, as well as in cancer survivors exposed to senescence-inducing treatments such as whole-brain irradiation.

## Introduction

The aging of populations across the globe—particularly in high-income countries—has led to a dramatic rise in the burden of chronic, age-related diseases, including cardiovascular disease [1–3], neurodegeneration and dementia [4, 5], cancer [6, 7], and frailty [8]. These conditions are not merely correlated with aging; rather, they are driven by the cellular and molecular mechanisms that underlie the aging process itself [9–11]. Understanding these fundamental mechanisms is essential for the development of interventions aimed at delaying or preventing age-related pathologies and extending healthspan.

While numerous cell-autonomous mechanisms—including mitochondrial dysfunction, oxidative stress, and genomic instability—have been implicated in aging [9–11], the synchronized functional decline observed across multiple organs suggests the existence of higher-order regulatory systems [12–15]. Growing evidence supports the role of central, cell non-autonomous mechanisms in coordinating systemic aging [15].

The brain, and particularly the hypothalamus, has emerged as a potential central regulator—or “pacemaker”—of systemic aging [14, 16–20]. As a key neuroendocrine hub, the hypothalamus integrates hormonal, metabolic, and immune signals and regulates organismal homeostasis throughout life [20–26]. Its functional deterioration with age has been linked to the emergence of diverse aging phenotypes, including metabolic dysregulation, reduced stress resilience, and physiological decline in multiple organ systems [14, 16, 18]. Recent studies suggest that hypothalamic aging is driven, at least in part, by chronic sterile inflammation and the accumulation of senescent cells [16, 20, 23, 25].

A hallmark of brain aging is the buildup of senescent cells—especially cerebromicrovascular endothelial cells and microglia—that adopt a senescence-associated secretory phenotype (SASP) [27–31]. These senescent cells release inflammatory cytokines, chemokines, and matrix-remodeling enzymes that not only disrupt local brain function but may also enter the circulation and influence distant peripheral organs.

While the presence of senescent cells in the aging brain is well established [30, 31], and brain senescence has been implicated in neuroinflammation and cognitive decline [27, 32], it remains unclear whether these cells can actively drive systemic aging. Notably, studies using heterochronic parabiosis[33] have shown that circulating factors from old animals can induce aging-like phenotypes in young partners—including in the heart, skeletal muscle, and vasculature—supporting the concept that blood-borne pro-geronic signals may contribute to peripheral aging [15, 34, 35]. However, direct experimental evidence demonstrating that brain senescence serves as a source of these systemic aging cues remains limited, representing a critical gap in the field.

In this study, we tested the hypothesis that senescence in the brain is sufficient to promote aging-like changes in distant organs. To this end, we employed a well-characterized model of whole-brain irradiation (WBI) in young mice, which our group has previously shown to induce widespread cellular senescence and neuroinflammation in the brain, recapitulating key features of natural brain aging. We focused on the heart, a peripheral organ known to be highly responsive to systemic inflammatory and metabolic cues, and performed transcriptomic profiling to assess the impact of brain senescence on cardiac gene expression. Our findings reveal a striking overlap between the cardiac transcriptomic signatures of WBI-treated young mice and naturally aged controls, supporting the hypothesis that brain senescence can orchestrate systemic aging via cell non-autonomous, likely blood-borne, mechanisms.

## Methods

### Experimental animals

**Fig. 1:**
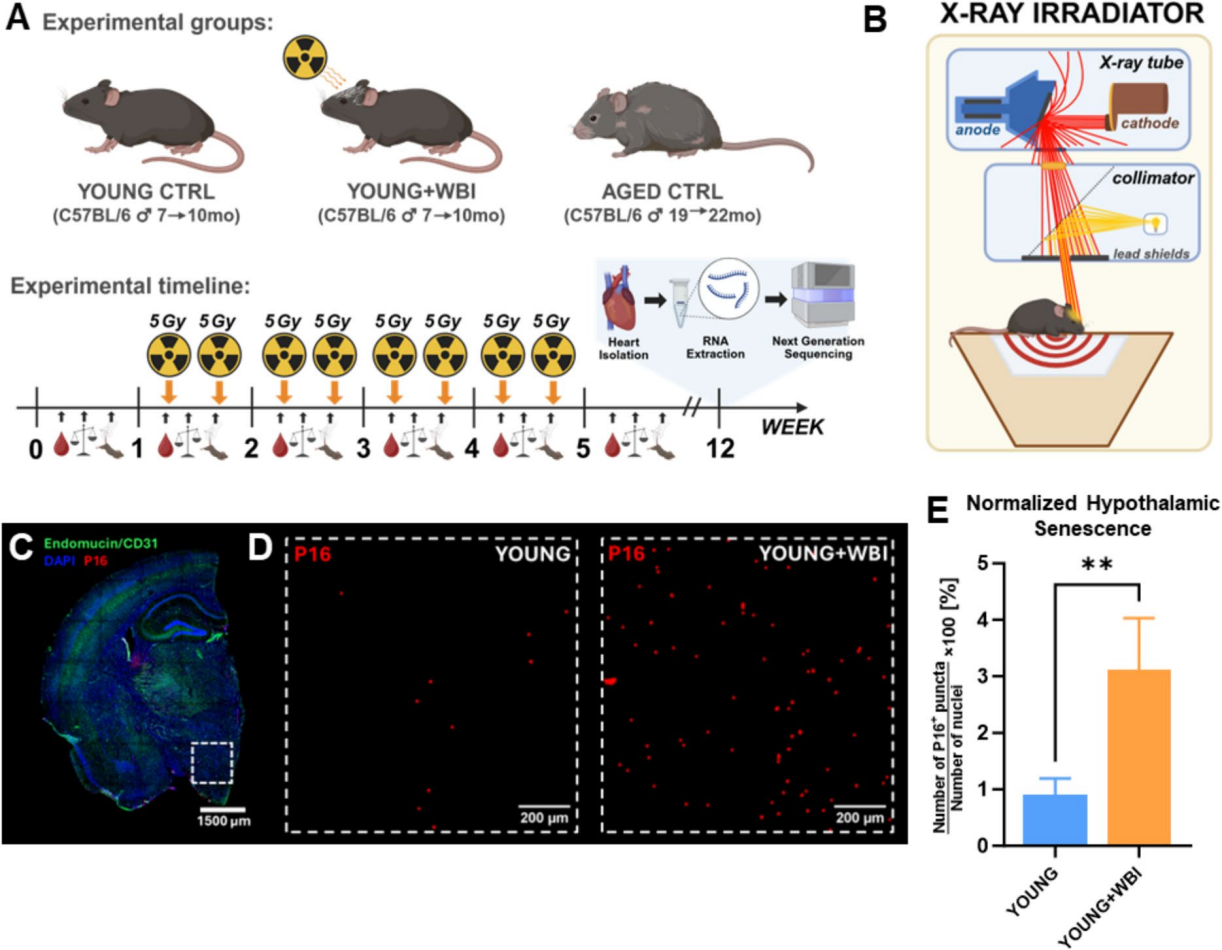
Experimental design and model validation. **A** Schematic of the experimental design. Three groups were included in this study: young control mice, young mice subjected to whole-brain irradiation (WBI), and naturally aged control mice. Baseline measurements of body weight, neuroscore, and complete blood counts were obtained 1 week prior to WBI (week 0). WBI was administered biweekly from weeks 1 to 4, delivering 5 Gy per session for a total dose of 40 Gy. Mice were monitored weekly during and for 5 weeks following the irradiation regimen to assess potential side effects. Two months after WBI, animals were euthanized, and hearts were collected for RNA isolation, cDNA library preparation, and next-generation sequencing. **B** Illustration of the X-ray irradiator used for WBI. The system is equipped with an X-ray tube and light-guided collimator, allowing precise targeting of the mouse brain while minimizing off-target radiation exposure to peripheral tissues. **C** Representative immunofluorescence image of a coronal brain section stained for vasculature (green, endomucin/CD31), senescent cells (red, p16), and nuclei (blue, DAPI). The white dashed box indicates the hypothalamic region used for higher magnification imaging and senescence quantification.** D** Representative images of the hypothalamus from young control and WBI-treated mice. A marked increase in p16-positive senescent puncta is evident in the hypothalamus following WBI. **E** Quantification of hypothalamic senescence. The number of p16-positive puncta was normalized to the number of nuclei. WBI induced a significant (~ 3.5-fold) increase in senescent cell burden compared to young controls (*p* < 0.01, Student’s *t*-test). Data are presented as mean ± SD

Young (7-month-old, *n* = 17) and aged (19-month-old, *n* = 9) male C57BL/6 mice (*Mus musculus*) were obtained from the National Institute on Aging (NIA) colony and housed in a specific pathogen-free animal facility at the University of Oklahoma Health Sciences Center (OUHSC). Animals were maintained under a 12:12 h light–dark cycle with ad libitum access to standard rodent chow (AIN-93G) and water in accordance with standard husbandry practices. One week prior to the first brain irradiation session, all mice were transferred from the Rodent Barrier Facility to the Conventional Rodent Facility at OUHSC, where they were housed under similar conditions. Nine young mice were randomly assigned to receive fractionated whole brain irradiation (WBI; two sessions per week for 4 weeks, Fig. 1A**)**. Following completion of the WBI protocol, irradiated mice were allowed to recover for 2 months. At the end of the recovery period, animals were humanely euthanized, and heart tissues were collected for transcriptomic analyses. Heart tissues from control mice were collected at 10 months (young) and 22 months (aged; Fig. 1A). All animal procedures were approved by the Institutional Animal Care and Use Committee (IACUC) at OUHSC and conducted in accordance with the National Institutes of Health Guide for the Care and Use of Laboratory Animals.

### Brain irradiation

Following 1 week of acclimation in the conventional facility, young mice were randomly assigned to either the WBI (*n* = 8) or control group (*n* = 9). During the irradiation procedures, mice were sedated using isoflurane anesthesia (Isoflurane USP, Cat# 029405, Covetrus, UK; 2.5% at a flow rate of 2.0 L/min). WBI was administered using an X-RAD320 X-ray irradiator (PRECISION, CT, USA) operating at 320 keV acceleration voltage and a dose rate of 0.8 Gy/min. The X-RAD 320 system is equipped with a collimator, which allows precise delivery of irradiation specifically to the brain, preventing off-target exposure to peripheral tissues (Fig. 1B). The WBI protocol consisted of eight irradiation sessions, delivered twice weekly over 4 weeks. Each session delivered a dose of 5 Gy, resulting in a cumulative total of 40 Gy (Fig. 1A). Following the final irradiation, mice were allowed to recover for 2 months.

### Tissue harvesting

At the experimental endpoint, animals were anesthetized with isoflurane and euthanized via intracardiac perfusion with ice-cold phosphate-buffered saline (PBS). Heart tissues from age-matched control mice were collected at 10 months of age (young controls) and 22 months of age (naturally aged controls), as indicated in Fig. 1A. We selected the heart as a representative peripheral organ because cardiac aging is a major contributor to morbidity and mortality in older adults and is known to be highly sensitive to systemic inflammatory and endocrine alterations. Moreover, cardiac function is intimately regulated by both vascular health and neuroendocrine inputs, making it a strategic target for studying interorgan aging dynamics. Following excision, the hearts were carefully trimmed to isolate the left ventricle (LV); the atria, aorta, large veins, pericardium, and pericardial fat were removed to ensure consistency across samples. The dissected LV tissue was then flash-frozen in liquid nitrogen and stored at –80 °C until further processing for transcriptomic analysis.

### Assessment of acute and systemic effects of WBI

To evaluate potential acute side effects of whole-brain irradiation, we monitored neurological function, body weight, and hematological parameters throughout the course of the experiment. Neurological function was assessed using a previously established NeuroScore assessment [36, 37], which evaluates six domains of neurobehavioral performance. Assessments were conducted weekly in each irradiated mouse, starting 1 week before the first irradiation session and continuing through the WBI regimen and for 5 weeks post-irradiation. The evaluated domains included (1) symmetry of limb movement when suspended by the tail, (2) body flexion and escape response (position awareness), (3) forelimb outstretching reflex, (4) spontaneous exploratory behavior, (5) climbing ability on a wire rack, and (6) response to whisker stimulation. Each test was scored either from 0 or 1 (severe deficit) to 3 (normal function), for a maximum total score of 18. In our study, WBI-treated mice maintained normal NeuroScores throughout the observation period, with no evidence of neurological deficits.

Body weight was also monitored weekly, beginning 1 week before irradiation and continuing through 5 weeks post-WBI. A transient, slight (~ 10%) weight loss was observed during the 4th and 5th week of the experimental timeline. Body weights returned to baseline values shortly after completion of the WBI regimen and remained stable until the experimental end-point, indicating that the WBI protocol was well-tolerated and observed transcriptomic changes were not caused by the acute effects of WBI (data not shown).

To assess the potential acute and long-term hematopoietic impact of WBI, we performed longitudinal blood analyses to monitor bone marrow activity. Fifty microliters of peripheral blood were collected weekly from the submandibular vein under light anesthesia (1.5% isoflurane at a 0.8 L/min flow rate). To minimize stress, blood sampling was alternated between two groups of mice, such that each individual mouse was sampled every 2 weeks, while group-level blood count data were collected weekly. Samples were drawn into K3-EDTA-coated tubes (Microvette 500 K3E, Cat#: 20.1341.102, Sarstedt, Germany). The automated five-part differential hematology analyses were performed by the Technical Services Unit of the Department of Comparative Medicine at OUHSC. Complete blood count parameters, including red blood cells, white blood cells, and platelets, were evaluated across the study period. While complete blood cell count, red blood cell count, and platelet number remained stable throughout the experimental timeline (data not shown), we observed a transient reduction in white blood cell counts during the weeks of the WBI protocol, which exhibited a trend toward significance (*p* = 0.05–0.33 vs. Week 0). These values returned to baseline following completion of the irradiation regimen, indicating that the observed leukopenia was mild, temporary, and reversible.

### Quantification of hypothalamic senescent cells

Quantification of senescent cells in the hypothalamus was performed using immunofluorescence staining for p16, a well-established marker of cellular senescence.

Brains were fixed in 4% paraformaldehyde for 24 h, followed by cryoprotection through sequential overnight incubations in 10%, 20%, and 30% sucrose (w/v in deionized water). Tissues were embedded in O.C.T. compound (Cat#: 4583, Sakura Finetek, USA) using embedding molds and frozen in a dry ice-chilled isopentane bath. Frozen brains were cryosectioned into 30-µm-thick coronal sections encompassing the hypothalamus. Sections were transferred to cryoprotectant solution (glycerol:ethylene glycol:1 × PBS:ddH₂O; 1:1:1:1, v/v/v/v) and stored at −20 °C until use.

On the day of staining, sections were rinsed twice with 1 × TBST [0.1% Tween-20 (Cat#: 1,610,781, Bio-Rad) in Tris-buffered saline] and twice with 1 × TBS. Blocking was performed at room temperature for 3 h in blocking buffer containing 0.5% Triton X-100 (Cat#: 85112, Thermo Fisher, USA), 5% bovine serum albumin (Cat#: A7030, Millipore Sigma), and 2.25% glycine (Cat#: G8898, Millipore Sigma) prepared in 10% normal goat serum (Cat#: 50062Z, Thermo Fisher, USA).

Sections were incubated overnight at 4 °C with primary antibodies diluted in blocking buffer: anti-CD31 (rat, 1:75, Cat#: BD550274, BD Biosciences), anti-endomucin (rat, 1:75, Cat#: MAB2624, Millipore Sigma), and anti-p16 (rabbit, 1:250, Cat#: AB211542, Abcam, UK). The following day, sections were washed twice with 1 × TBST and twice with 1 × TBS, then incubated for 3 h at room temperature, protected from light, with the following secondary antibodies: Alexa Fluor 488-conjugated anti-rat goat antibody (1:500, Cat#: A11006, Thermo Fisher) and Alexa Fluor 647-conjugated anti-rabbit goat antibody (1:500, Cat#: A21244, Thermo Fisher).

Nuclei were counterstained with DAPI (2.86 µM, Cat#: D1306, Thermo Fisher) for 5 min at room temperature, protected from light. After two final washes in 1 × TBST and 1 × TBS, sections were mounted onto glass slides (Cat#: 16,004–406, VWR) using ProLong Diamond Antifade Mountant (Cat#: P36930, Thermo Fisher) and coverslipped. Slides were cured overnight at room temperature in the dark. Control sections stained with secondary antibodies only or no primary/secondary antibodies were processed in parallel to assess background fluorescence.

Imaging was performed using a Leica Stellaris 8 confocal microscope equipped with an HC PL APO 10 ×/0.40 CS2 dry objective, white light laser, and 405-nm diode laser. Emission detection was carried out with Leica HyD detectors: S1 (425–504 nm, DAPI), S2 (504–663 nm, Alexa Fluor 488), and X3 (663–834 nm, Alexa Fluor 647). Hypothalamic regions were anatomically identified using the Allen Brain Atlas. Images were acquired with dimensions of 1107.14 µm × 1107.14 µm × 30 ± 10 µm (XYZ) and pixel sizes of 1.082 µm (X/Y) and 2.409 µm (Z). All imaging was performed using identical acquisition settings across samples.

Images were uniformly processed in ImageJ (v1.53t, NIH, USA). Z-stacks were converted to maximum intensity projections. Senescent puncta were segmented by thresholding the Alexa Fluor 647 channel (threshold: 100–255) and quantified using the Analyze Particles tool (size, 2–100 µm^2^; circularity, 0.0–1.0). The number of p16-positive puncta corresponding to senescent cells was normalized to the number of nuclei, segmented using the DAPI channel (threshold, 60–100; particle size, 20–100 µm^2^; circularity: 0.3–1.0). Quantification was performed on 2–3 hypothalamic sections per animal, and data were averaged to generate one biological replicate per mouse. Results are presented as the number of p16-positive puncta per 100 nuclei. Representative images and quantification results are shown in Fig. 1C–E. For visualization purposes, p16-positive puncta were enlarged using the Maximum Filter (5-pixel radius) in ImageJ.

### RNA isolation, cDNA library preparation, and next-generation sequencing

Total RNA was isolated from frozen left ventricular heart tissue samples using the RNeasy Fibrous Tissue Mini Kit (cat#: 74,704, QiaGEN, Germany) according to the manufacturer’s instructions. Briefly, 30 mg of tissue was homogenized in RLT lysis buffer using mechanical disruption. The lysates were centrifuged at 1000* g* for 10 min at 4 °C, and the resulting supernatants were transferred to clean microcentrifuge tubes. RNA was then isolated using the QIAcube Connect MDx system (QIAGEN, Germany), a fully automated platform utilizing spin column–based extraction technology. The quantity and integrity of the extracted RNA were assessed using both a NanoDrop Microvolume Spectrophotometer and an Agilent TapeStation system. The mean RNA Integrity Number (RIN) values were as follows: Young = 7.3 ± 1.3, Young + WBI = 8.9 ± 0.3, and Aged = 7.9 ± 1.4 (mean ± SD), indicating optimal RNA quality for downstream transcriptomic analysis.

Following quality assessment, cDNA library preparation was performed at the OUHSC Genomics Core Facility. Messenger RNA was enriched using the NEBNext Poly(A) mRNA Magnetic Isolation Module (Cat# E7490S, New England Biolabs, USA), and libraries were constructed using the xGen Broad Range RNA Library Prep Kit (Cat# 10,009,866, Integrated DNA Technologies, USA) in accordance with the manufacturer’s protocol. Library quality and concentration were verified using the Agilent TapeStation. Equimolar amounts of indexed libraries were pooled and sequenced on an Illumina NextSeq 2000 platform using sequencing-by-synthesis chemistry to generate 151 bp paired-end reads.

### RNA-Seq data processing and differential expression analysis

Raw sequencing reads were assessed for quality using FastQC (v0.11.9) and MultiQC (v1.12) to generate an aggregated quality report. Adapter sequences and low-quality reads (Phred score < 20) were removed using *Trimmomatic* (v0.39). Transcript abundance was quantified by pseudo-alignment to the mouse transcriptome (GRCm39) using Kallisto (v0.46.2).

Sample-level quality control and outlier detection were performed after quantification. Estimated counts and transcript lengths were imported and summarized to the gene level using the *tximport* R package (v1.28.0) [38]. Lowly expressed genes were filtered out, retaining only genes with at least 10 raw counts in at least 3 samples. Principal component analysis (PCA) was conducted using the prcomp R function to identify and exclude potential outlier samples visually.

Differential expression analysis was performed using the DESeq2 R package (v1.44.2) [39]. DESeq2 utilizes a negative binomial generalized linear model (GLM) to model count data and Wald statistics for hypothesis testing. The raw counts, imported via *tximport*, were directly used as input. Significantly differentially expressed (DE) genes were identified based on an adjusted *p*-value (Benjamini-Hochberg False Discovery Rate, FDR; *p*) < 0.05 and an absolute logarithmic fold change (|FC|) > 1.5. Gene annotations (Entrez IDs and gene symbols) were retrieved from Ensembl IDs using the org.Mm.eg.db R package (v3.19.1) for comprehensive annotation querying from the Ensembl database.

### Functional enrichment analysis

Gene Ontology (GO) terms (biological process, molecular function, cellular component), KEGG pathways, Reactome pathways, and Hallmark pathways associated with the differentially expressed gene sets were collected and analyzed. Gene set enrichment analysis (GSEA) was performed using the clusterProfiler R package (v4.8.3) [40]. For GSEA, genes were ranked by their weighted log2 Fold Change (FC * −log10(*p*)) values.

Enrichment maps for visualizing the relationships between enriched pathways were created using the EnrichmentMap plugin (v3.5.0) [41] within Cytoscape (v3.7.0) [42]. Changes in transcription factor (TF) activity were predicted using the decoupleR R package (v2.8.0) [43] by leveraging the DoRothEA mouse regulon [44]. Results of decoupleR were also validated by upstream regulator analysis available in the ingenuity pathway analysis (IPA, QIAGEN) software.

### Upstream regulator analysis

To identify upstream regulators that may account for the observed gene expression changes in our samples, we performed upstream regulator analysis (URA) using the ingenuity pathway analysis (IPA, QIAGEN) software package [45]. IPA is a widely used commercial bioinformatics platform that integrates differentially expressed genes with known biological functions, signaling pathways, and regulatory networks curated in the Ingenuity Knowledge Base. This knowledge base is a proprietary, manually curated collection comprising nearly 5 million findings derived from peer-reviewed scientific literature and reputable third-party databases. The URA algorithm predicts potential upstream transcriptional regulators based on the direction and magnitude of observed gene expression changes, providing activation *z*-scores and overlap *p*-values to estimate the likelihood and directionality of regulator activity. Additional details on IPA methodology are available at: http://qiagen.force.com/KnowledgeBase/.

### Statistical analyses

All statistical analyses were performed using GraphPad Prism (v10.0, GraphPad Software, San Diego, CA) and R software (v4.3.2) with relevant packages as described. Group differences in hypothalamic senescence burden were assessed using unpaired, two-tailed Student’s *t*-tests. Longitudinal data, including body weight and hematological parameters, were analyzed using repeated measures one-way ANOVA with post-hoc comparisons to baseline (week 0) when appropriate. Transcriptomic differential expression analyses were conducted using the DESeq2 package, employing a negative binomial generalized linear model with Wald statistics and Benjamini-Hochberg adjustment for multiple testing. Gene set enrichment significance was determined using permutation-based approaches implemented in the clusterProfiler package. Correlation analyses between gene expression profiles were performed using Pearson’s correlation coefficient. Statistical significance was defined as *p* < 0.05 unless otherwise specified. Data are presented as mean ± standard deviation (SD).

## Results

### Brain irradiation induces aging-like transcriptomic changes in the heart

To evaluate whether brain senescence can exert systemic effects on peripheral organs, we employed a well-established model of WBI in young mice. This model induces cellular senescence specifically in the brain and has previously been shown by our group to mimic several key features of natural brain aging [46, 47]. NeuroScore analysis demonstrated that WBI did not impair neurological condition during or after the irradiation protocol (data not shown). Similarly, no significant differences in body weight, red blood cell count, platelet count, or white blood cell count were observed between WBI-treated mice and young controls throughout the experimental timeline, indicating that the WBI protocol did not induce overt systemic toxicity. Two months after WBI, hearts were collected from these mice for transcriptomic profiling and compared with age-matched non-irradiated controls and naturally aged mice.

We first confirmed that WBI effectively induced cellular senescence in the hypothalamus, a brain region implicated in the regulation of systemic aging. Immunofluorescence staining for p16, a widely used marker of senescence, revealed a significant accumulation of senescent cells in the hypothalamus of WBI-treated mice compared to young controls (Fig. 1C–E). Quantification of p16-positive puncta, normalized to the number of nuclei, demonstrated an approximately 3.5-fold increase in hypothalamic senescence following WBI (*p* < 0.01, Student’s *t*-test). These findings confirm that our WBI protocol reliably induces hypothalamic senescence, providing a mechanistic basis to explore how brain aging may influence peripheral organs.

**Fig. 2:**
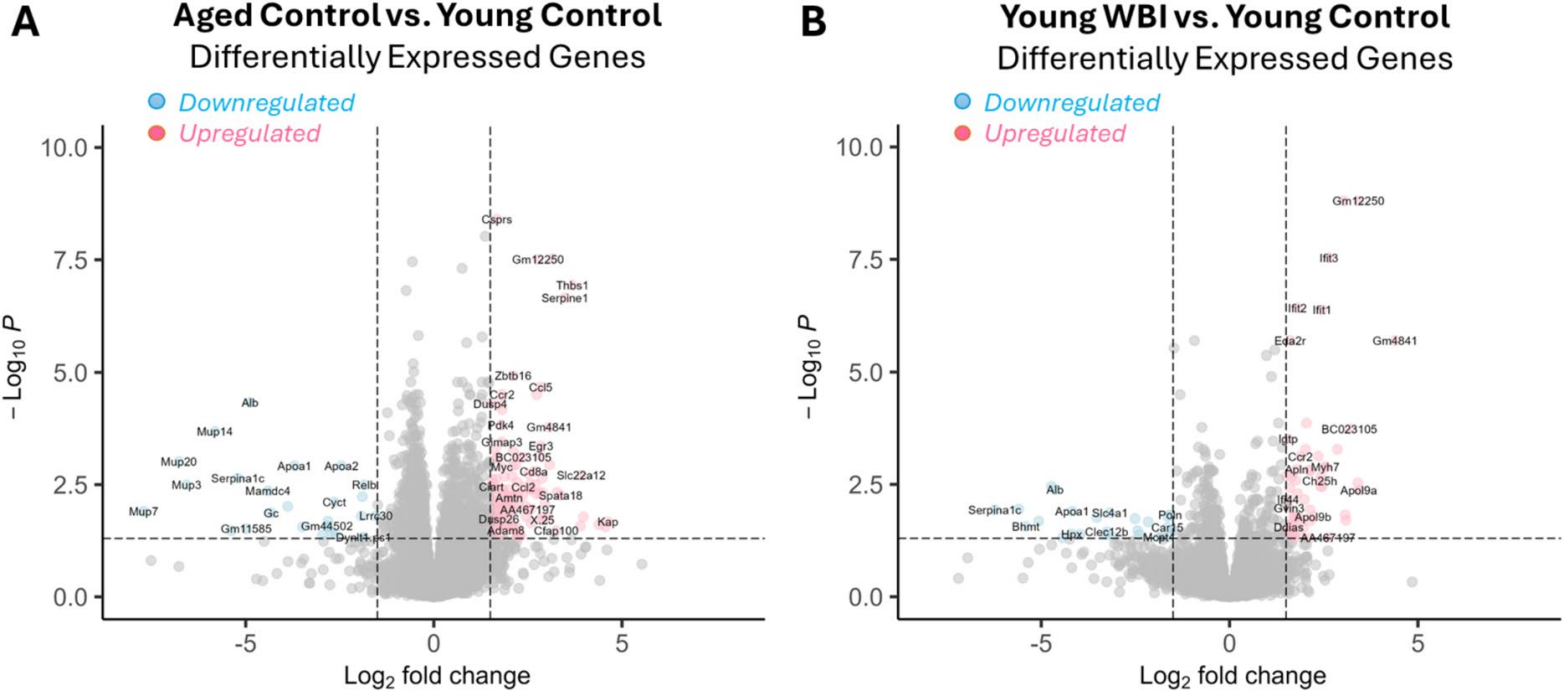
WBI-induced brain senescence induces aging-like changes in the cardiac transcriptome. **A** Volcano plot showing differentially expressed genes (DEGs) in the hearts of aged control mice compared to young controls. **B** Volcano plot showing DEGs in the hearts of young mice 2 months after whole-brain irradiation (WBI), compared to age-matched young controls. In both panels, the –log₁₀(*p*-value) is plotted against the log₂ fold change in gene expression. Each dot represents an individual gene. Significantly upregulated genes are shown in red, and significantly downregulated genes are shown in blue (FDR-adjusted *p*-value < 0.05 and fold change ≥ 1.5 or ≤ 0.67). Selected DEGs relevant to inflammation, ECM remodeling, and cardiac stress are labeled. The plots demonstrate that WBI-induced brain senescence in young mice leads to transcriptional changes in the heart that resemble those observed during natural aging. *n* = 8–9 mice per group

Transcriptomic profiling of the heart revealed substantial gene expression remodeling in both aged hearts and those from WBI-treated young mice, relative to young controls. As shown in Fig. 2A, aged mice exhibited a large number of differentially expressed genes (DEGs), with significant upregulation and downregulation of transcripts implicated in inflammation, extracellular matrix (ECM) remodeling, and cardiac stress. Similarly, hearts from WBI-treated mice displayed a robust DEG profile (Fig. 2B), including upregulation of inflammatory markers such as *Ccl5* and *Ifit3*, ECM-related genes such as *Serpine1* and *Adam8*, and stress-associated genes including *Myh7* and *Apol9*. Together, these patterns imply that inducing senescence through brain-specific irradiation is sufficient to drive aging-like transcriptional remodeling in the heart.

### Concordant transcriptomic shifts between aging and brain irradiation

**Fig. 3:**
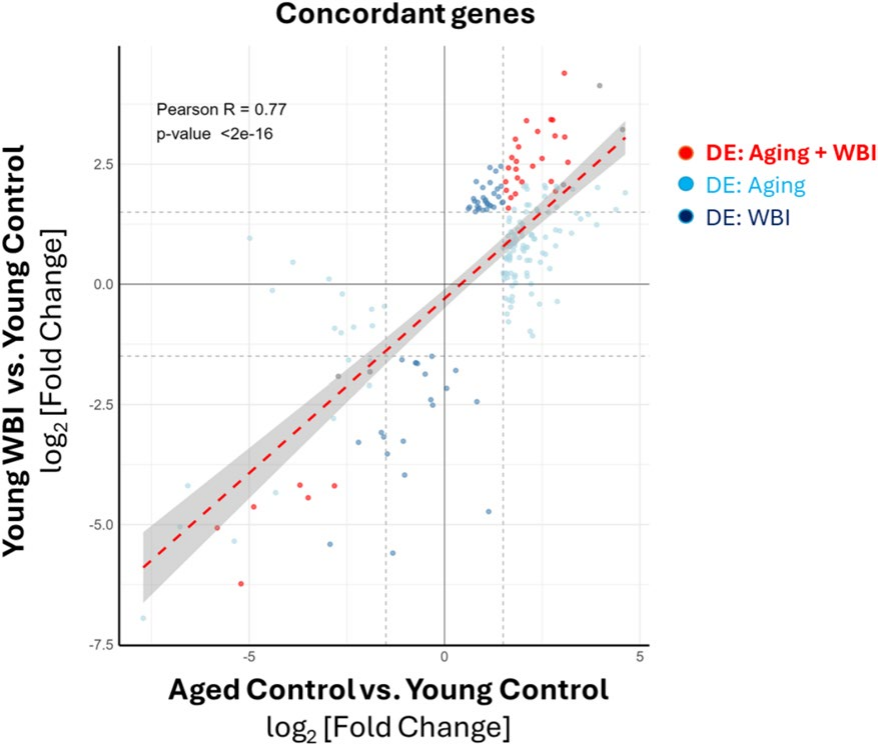
WBI-induced brain senescence promotes aging-like changes in the cardiac transcriptome: identification of concordant gene regulation patterns. Scatter plot comparing log₂ fold changes in gene expression in the hearts of young mice subjected to whole-brain irradiation (WBI) versus age-matched young controls (y-axis) and aged controls versus young controls (x-axis). Each point represents a gene. Red symbols indicate concordant differentially expressed (DE) genes that are significantly regulated in both comparisons (FDR-adjusted *p*-value < 0.05), with the same direction of effect. Light blue and dark blue points represent genes that are concordantly regulated (i.e., with the same direction of fold change) but only reach statistical significance in the aging (light blue) or WBI (dark blue) comparison, respectively. The dashed red line and shaded area indicate the linear regression fit (Pearson *R* = 0.77, *p* < 2e–16), demonstrating a strong positive correlation between age-related and WBI-induced transcriptomic remodeling in the heart. These results suggest that WBI-induced brain senescence recapitulates key features of cardiac aging at the transcriptomic level. *n* = 8–9 mice per group

To directly compare the effects of natural aging and WBI-induced brain senescence on cardiac gene expression, we analyzed the relationship between the heart transcriptomes of naturally aged mice and young mice subjected to WBI. Correlation analysis of log₂ fold changes across the transcriptomes revealed a strong linear association between the two conditions (*R* = 0.77, *p* = 2e–16; Fig. 3), indicating that many genes were regulated in the same direction. Genes upregulated in aged hearts also tended to be upregulated in WBI-treated hearts, and vice versa, suggesting that brain senescence can drive aging-like transcriptional changes in the heart.

To avoid excluding biologically meaningful overlap due to overly strict significance thresholds, we adopted a more inclusive analytical strategy to identify what we refer to as “pro-geronic shifts.” Specifically, we retained genes that were differentially expressed (adjusted *p* < 0.05 and absolute log₂ fold change ≥ 0.58) in one condition and showed a consistent direction of change with at least a 1.5-fold difference (|log₂FC| ≥ 0.58) in the other, even if not meeting significance there. This approach captured concordant transcriptomic remodeling beyond the intersection of statistically significant DEGs. Using this strategy, we identified 162 genes that were upregulated and 49 genes that were downregulated in both aged and WBI-treated hearts. No genes showed opposing regulation between the two conditions. These results provide strong evidence that WBI-induced brain senescence recapitulates a subset of the molecular hallmarks of natural cardiac aging. Together, these findings support the concept that localized senescence in the brain is sufficient to initiate systemic, pro-aging transcriptional programs in peripheral organs such as the heart, likely through cell non-autonomous mechanisms involving endocrine or inflammatory signaling.

### Overlapping gene sets and aging pathways are regulated by brain senescence

**Fig. 4:**
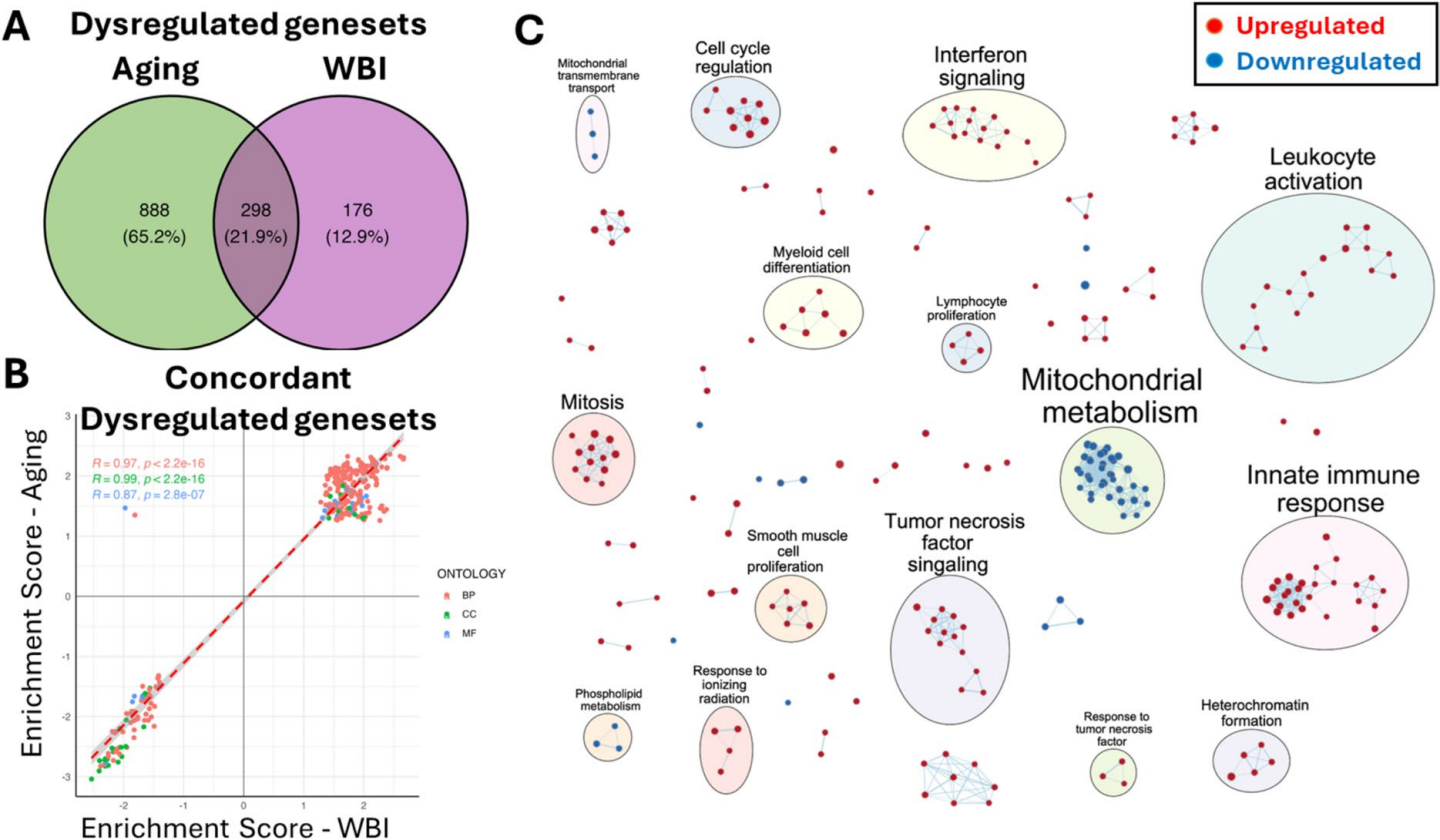
Gene set enrichment analysis reveals shared biological programs in the mouse heart affected by aging and WBI-induced brain senescence. **A** Venn diagram showing the overlap between gene sets significantly dysregulated in the hearts of aged mice and WBI-treated young mice. A total of 298 gene sets were concordantly dysregulated across both comparisons, representing 21.9% of all significantly altered pathways. **B** Correlation of enrichment scores for gene sets dysregulated in both aging and WBI conditions. Each point represents a gene set, categorized by ontology: biological process (BP), cellular component (CC), or molecular function (MF). A strong correlation was observed across all categories (*R* = 0.87–0.99, *p* < 2.2e–16), indicating a shared biological signature. **C** Network visualization of the top concordantly dysregulated gene sets. Nodes represent enriched pathways and are colored by directionality: red for upregulated and blue for downregulated in both aging and WBI hearts. Clusters highlight functionally grouped biological processes, including mitochondrial metabolism, interferon signaling, ECM remodeling, mitotic regulation, and immune activation. These results demonstrate that WBI-induced brain senescence recapitulates key aging-associated transcriptional programs in the heart

To determine whether shared biological pathways underlie the observed transcriptomic changes, we performed gene set enrichment analysis (GSEA) across the WBI and aging comparisons. We identified 298 gene sets that were significantly and concordantly dysregulated in both groups (Fig. 4A). These gene sets encompassed several evolutionarily conserved mechanisms of aging, including mitochondrial dysfunction, immune activation, interferon and TNF signaling, ECM remodeling, and cell cycle regulation.

**Fig. 5:**
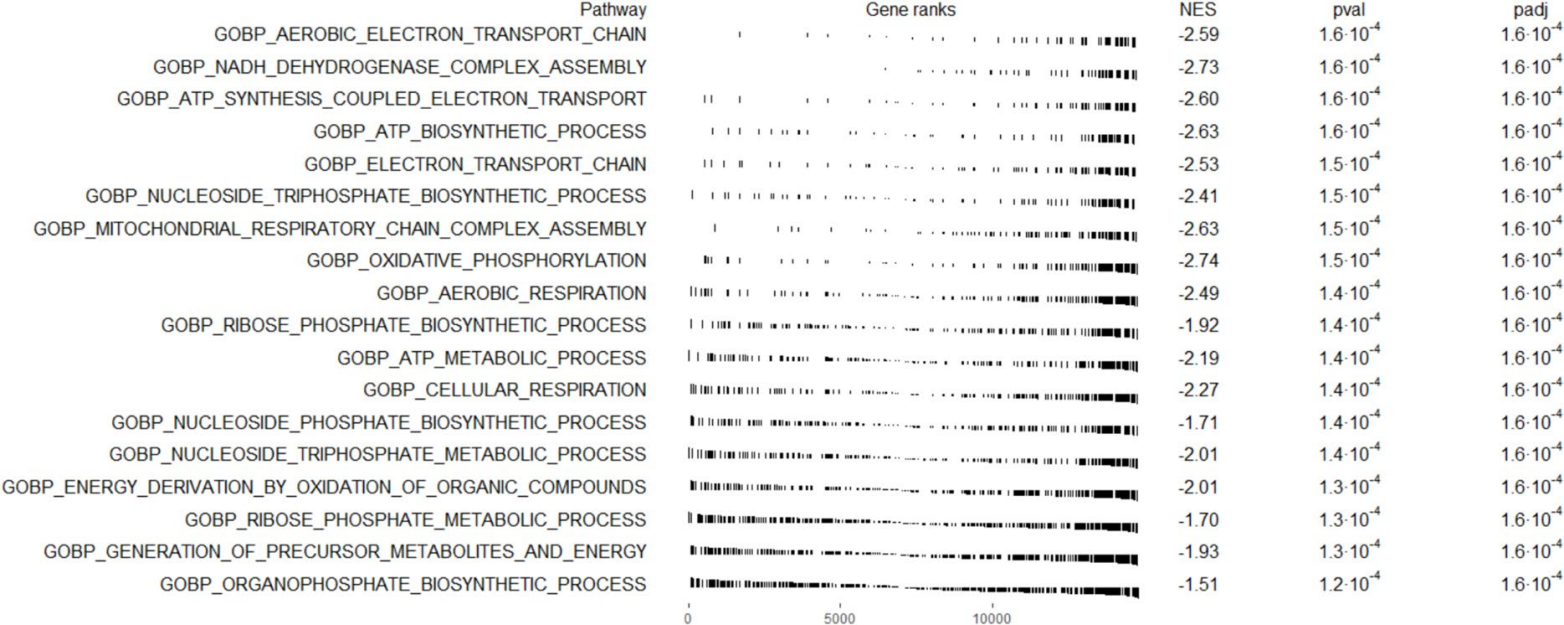
WBI-induced brain senescence induces coordinated downregulation of mitochondrial and energy metabolism pathways in the heart. Gene set enrichment analysis (GSEA) of transcriptomic profiles from hearts of WBI-treated young mice compared to young controls. Shown are gene sets significantly downregulated in the WBI group, all related to mitochondrial function, oxidative phosphorylation, electron transport chain activity, and ATP biosynthesis. The plot displays normalized enrichment scores (NES), raw *p*-values, and adjusted *p*-values (padj) for each pathway. Negative NES values indicate a coordinated reduction in the expression of genes associated with these metabolic processes. The gene ranks along the x-axis represent the position of genes within the ranked list based on differential expression between WBI-treated and control hearts, with black vertical lines indicating the location of genes belonging to each pathway. These findings demonstrate that WBI-induced brain senescence is associated with a robust transcriptional signature of impaired mitochondrial metabolism in the heart, a key feature of cardiac aging

Strikingly, enrichment scores for these gene sets showed an extremely high correlation across the two conditions (*R* = 0.87–0.99), indicating that not only individual genes but also entire biological programs are similarly affected by WBI-induced brain senescence and natural aging (Fig. 4B). Enrichment map analysis of the top overlapping gene sets further highlighted common biological themes, particularly involving innate immune signaling, metabolic dysfunction, and structural remodeling of cardiac tissue (Fig. 4C). These findings demonstrate that brain irradiation-induced senescence triggers a systemic cascade that recapitulates transcriptomic aging signatures in the heart, reinforcing the concept that the brain functions as a master regulator of organismal aging via cell non-autonomous mechanisms. While direct markers of cellular senescence were not robustly upregulated in the heart, the strong activation of SASP-associated pathways and matrix remodeling genes is consistent with a paracrine pro-senescent milieu. These findings raise the possibility of secondary senescence induction in the heart, which warrants further investigation using senescence-specific reporters or staining techniques. To further delineate the biological processes affected in the heart following WBI-induced brain senescence, we performed GSEA with a focus on pathways related to energy metabolism. As shown in Fig. 5, we observed a coordinated downregulation of gene sets involved in mitochondrial function, oxidative phosphorylation, and ATP biosynthesis in hearts from WBI-treated mice compared to young controls. Among the most significantly suppressed pathways were aerobic respiration, mitochondrial electron transport chain activity, and nucleoside triphosphate biosynthesis. These transcriptional changes are indicative of impaired mitochondrial energy metabolism—a recognized hallmark of cardiac aging—and suggest that brain senescence is sufficient to trigger aging-like mitochondrial dysfunction in peripheral organs such as the heart.

### Predicted activation of inflammatory and stress-related transcription factors in the hearts of WBI-treated mice

**Fig. 6:**
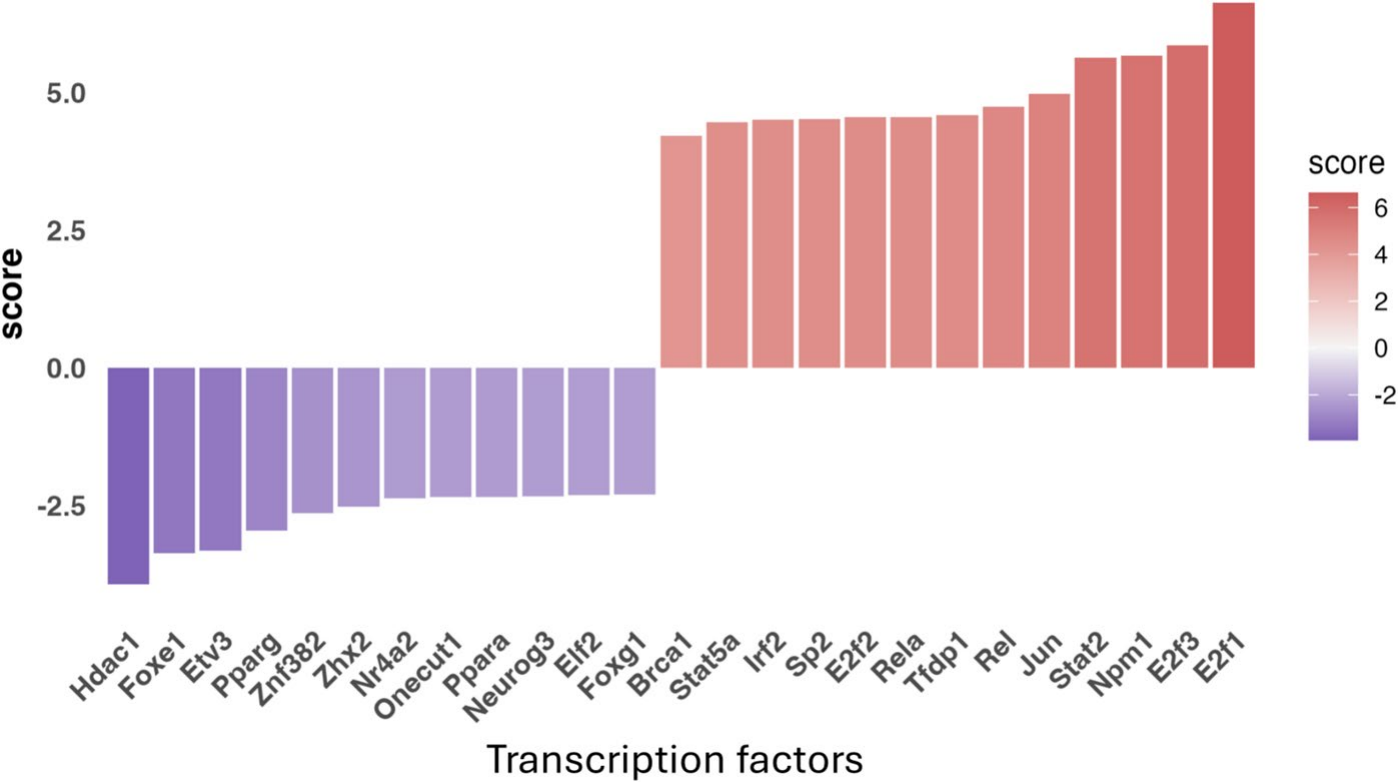
Predicted upstream transcriptional regulators mediating the effects of WBI-induced brain senescence on the cardiac transcriptome. Results from the decoupleR analysis identifying transcription factors (TFs) predicted to be significantly activated (red bars) or inhibited (purple bars) in the hearts of WBI-treated mice based on observed transcriptomic changes. The activation scores quantify the direction and magnitude of predicted TF activity, reflecting concordance between our dataset and established target gene signatures curated from prior experimental evidence. Many of the activated regulators are key effectors of innate immunity, interferon signaling, and cellular stress responses, suggesting engagement of canonical inflammatory pathways. In contrast, TFs associated with metabolic homeostasis and chromatin regulation were predicted to be suppressed. These findings support a model in which brain-derived senescence-associated signals drive a pro-inflammatory and metabolically repressive transcriptional program in the heart

To investigate upstream signaling mechanisms that may drive the transcriptomic remodeling observed in the hearts of WBI-treated mice, we performed upstream regulator analysis using the decoupleR R package (v2.8.0). This analysis predicted significant activation of multiple transcription factors (TFs) associated with inflammation, immune activation, and cellular stress responses (Fig. 6). Prominently upregulated TFs included RELA (NF-κB p65), REL, STAT2, JUN, IRF2, and members of the E2F family (notably E2F1 and E2F3), all of which are canonical mediators of pro-inflammatory cytokine and interferon signaling. Notably, these TFs are downstream targets of SASP components such as IL-1β, IL-6, and type I interferons, suggesting that circulating inflammatory mediators released from the senescent brain may engage transcriptional stress pathways in the heart.

In contrast, several transcription factors associated with metabolic regulation, chromatin remodeling, and mitochondrial function—including Hdac1, Ppara, Pparg, and Foxe1—were predicted to be inhibited. This reciprocal pattern of transcriptional activation and suppression reflects the emergence of a pro-geronic gene expression program, reinforcing the hypothesis that WBI-induced brain senescence alters the systemic environment in a way that accelerates aging in peripheral organs.

To confirm these findings, we conducted an independent upstream regulator analysis using ingenuity pathway analysis (IPA). The results demonstrated strong concordance with the transcription factors identified by decoupleR (Table 1), supporting the robustness of our conclusions. Consistent with the decoupleR predictions, IPA revealed broad activation of upstream regulators in the hearts of WBI-treated mice, including multiple interferons (IFN-α, IFN-β, IFN-γ), pro-inflammatory cytokines (IL-6, IL-1β, TNF), and innate immune sensors (STING1, CGAS, TLR3, TLR4, RIG-I). These signatures implicate systemic activation of interferon and pattern recognition receptor pathways, likely driven by circulating SASP factors or damage-associated molecular patterns released from the senescent brain. In parallel, several metabolic and chromatin regulators—such as SIRT1, HNF4A, PRDM16, and PPARA—were predicted to be inhibited, reinforcing the emergence of a pro-aging transcriptional program in cardiac tissue. Importantly, the inhibition of these factors also suggests impaired hypothalamic function and suppression of the GH/IGF-1 axis, a key neuroendocrine pathway in systemic aging regulation. SIRT1, PPARA, PRDM16, and HNF4A are well-established downstream targets of IGF-1 signaling that regulate mitochondrial biogenesis, metabolic homeostasis, and cardioprotection. Their suppression, alongside the activation of inflammatory and interferon pathways known to antagonize somatotropic signaling, indicates a potential systemic decline in anabolic tone. These transcriptomic changes are consistent with clinical observations of reduced circulating IGF-1 levels in patients receiving cranial radiotherapy, supporting the idea that localized brain injury can impair hypothalamic-pituitary output and accelerate endocrine aging. Thus, our findings raise the possibility that brain senescence contributes to peripheral tissue aging not only via inflammatory SASP factors but also by disrupting neuroendocrine homeostasis through suppression of the GH/IGF-1 axis.

**Table 1:** Key upstream regulators predicted to mediate the cardiac effects of WBI-induced brain senescence. Summary of representative upstream regulators identified through Ingenuity Pathway Analysis (IPA) in the hearts of WBI-treated mice. Regulators are grouped by functional category, with predicted activation or inhibition based on transcriptomic signatures. Inflammatory cytokines and pattern recognition receptors reflect systemic immune activation, while SASP-associated transcription factors (TFs) mediate stress-responsive gene expression. Inhibited metabolic regulators and downstream effectors of IGF-1 signaling suggest suppression of anabolic and mitochondrial programs, consistent with endocrine features of accelerated aging

Category	Upstream regulator	Type	Predicted activation
Inflammatory cytokines	IL-6, IL-1β, TNF, IFNG, IFNB1	Cytokine	Activated
Pattern recognition receptors	TLR4, TLR3, RIG-I, STING1, CGAS	PRR/Sensor	Activated
SASP-associated TFs	RELA, STAT1/2, IRF7, E2F1/3	Transcription factor	Activated
Metabolic regulators	SIRT1, HNF4A, PRDM16, PPARA	Transcription factor	Inhibited
Endocrine pathway	IGF-1 target regulators (SIRT1, PRDM16, HNF4A)	Downstream effectors	Inhibited

## Discussion

The present study provides direct experimental evidence that senescence confined to the brain is sufficient to trigger aging-like transcriptional remodeling in a peripheral organ. Using a well-characterized model of WBI to induce senescence and neuroinflammation in young mice [46–48], we observed an interesting overlap between the cardiac transcriptomic profiles of brain-irradiated animals and those of naturally aged controls. These findings support a model in which cellular senescence in the brain exerts systemic, cell non-autonomous effects that accelerate aging processes in distant organs via circulating mediators.

Our analysis revealed that WBI-induced brain senescence triggers systemic changes that recapitulate key aspects of the cardiac aging transcriptome, with dozens of genes showing concordant regulation in both naturally aged hearts and hearts of WBI-treated mice. Many of these genes are involved in evolutionarily conserved mechanisms of aging, including mitochondrial dysfunction [49], immune activation, interferon signaling, and ECM remodeling—pathways that are also widely implicated in age-related cardiovascular diseases. These transcriptomic shifts are highly consistent with aging signatures described in prior studies of cardiac aging [50–56], underscoring the biological relevance of the systemic impact induced by brain senescence. Collectively, these findings reinforce the hypothesis that local senescence, even when confined to the central nervous system, can reshape the peripheral transcriptome in a manner that phenocopies natural aging [15, 57–59].

In addition to promoting inflammatory and stress-related transcriptional remodeling in the heart, our data demonstrate that WBI-induced brain senescence is associated with a striking suppression of mitochondrial energy metabolism pathways in cardiac tissue. The coordinated downregulation of gene sets involved in oxidative phosphorylation, electron transport chain activity, and ATP biosynthesis closely mirrors transcriptional features of naturally aged hearts and aligns with previous studies linking mitochondrial dysfunction to age-related cardiac decline [60–62]. These findings suggest that brain senescence not only triggers inflammatory and extracellular matrix remodeling but also contributes to impaired mitochondrial function in peripheral organs, potentially through circulating factors or neuroendocrine signaling. This highlights the multifaceted systemic impact of brain aging and reinforces the need to consider mitochondrial pathways as downstream targets of brain-periphery communication in aging.

Our findings build upon, and extend, previous work using heterochronic parabiosis models. Studies in which young mice are surgically connected to aged partners have demonstrated that circulating factors in old blood can promote aging-like phenotypes in multiple tissues, including the vasculature, heart, skeletal muscle, and brain [15, 34, 35]. While these studies implicate blood-borne pro-geronic signals in the systemic propagation of aging, they do not identify the cellular origin of such factors. The current study points to brain senescence—and potentially hypothalamic senescence in particular—as a mechanistically relevant source of circulating pro-aging signals.

Senescent cells in the brain, particularly microglia and endothelial cells [30], are known to adopt a pro-inflammatory SASP, releasing cytokines, chemokines, proteases, and extracellular vesicles into the brain microenvironment. While originally described as mediators of local tissue dysfunction [27], it is increasingly appreciated that SASP factors may enter the circulation and modulate peripheral physiology. In this context, our data provide compelling evidence that brain-derived SASP components or secondary neuroendocrine changes induced by senescence can reprogram distant organ gene expression, potentially promoting maladaptive tissue remodeling and functional decline.

To gain mechanistic insight into the signaling pathways underlying the observed transcriptomic remodeling in the heart, we performed upstream regulator analysis using the decoupleR R package, followed by validation with IPA. This analysis revealed robust activation of several transcription factors associated with inflammation, innate immune signaling, and cellular stress responses. These transcription factors are well-characterized downstream effectors of pro-inflammatory cytokines (e.g., IL-1β, IL-6, and TNF-α) and type I interferons—key components of the SASP. Their activation in the heart of WBI-treated animals suggests that circulating SASP factors, released from senescent brain cells, may act systemically to induce stress-responsive transcriptional programs in peripheral organs. Importantly, the predicted inhibition of key metabolic and chromatin regulators—including SIRT1, PRDM16, HNF4A, and PPARA—suggests that the systemic inflammatory milieu may also suppress anabolic, mitochondria-supportive programs in peripheral tissues. Several of these regulators are also known targets of IGF-1 signaling, suggesting that WBI-induced brain injury may perturb hypothalamic control of the GH/IGF-1 axis. This adds a neuroendocrine dimension to the systemic aging model and is consistent with clinical reports of reduced IGF-1 levels and adverse cardiac remodeling in patients following cranial radiotherapy. Together, these findings reinforce the hypothesis that localized brain senescence can exert widespread influence through both inflammatory and endocrine pathways, promoting aging-like changes in distant organs such as the heart.

In parallel with the rise of pro-geronic signaling from the aging brain, aging is characterized by a decline in brain-derived anti-geronic factors that normally support systemic health [15]. One of the most prominent examples is the age-associated suppression of the GH/IGF-1 axis, regulated in part by hypothalamic output [19, 63–71]. Reduced secretion of hypothalamic growth hormone-releasing hormone (GHRH) during aging leads to decreased circulating GH and IGF-1 levels, which have been linked to diminished cardiac performance, muscle wasting, and increased vulnerability to frailty [72–75]. Notably, prior clinical and experimental studies have shown that brain irradiation, including WBI protocols similar to those used in our model, can disrupt hypothalamic function and suppress the GH–IGF-1 axis [76–83]. In pediatric cancer survivors, cranial irradiation is a well-recognized cause of growth hormone deficiency and chronically reduced IGF-1 levels, contributing to impaired growth, metabolic dysfunction, and early onset of aging-related comorbidities [76–79, 81, 82]. Preclinical studies similarly demonstrate that WBI in young animals reduces hypothalamic GHRH expression and systemic IGF-1 levels, underscoring the sensitivity of this neuroendocrine axis to radiation-induced brain injury [84]. This dual shift—from a decline in protective neuroendocrine outputs to an increase in inflammatory and matrix-modifying signals (e.g., SASP)—may synergistically reprogram peripheral organs toward an aged phenotype. While this pilot study was not designed to assess neuroendocrine outputs, future work using this model should include longitudinal measurements of GH, IGF-1, and other brain-derived endocrine factors to better characterize the interplay between senescence-associated inflammation and hormonal decline in driving systemic aging.

These findings may have particular relevance in two key populations. First, individuals with unhealthy aging trajectories—marked by obesity, unhealthy diets, environmental exposures, chronic stress, and sedentary lifestyle—are known to experience accelerated brain aging and hypothalamic inflammation [85–89]. Our data suggest that brain senescence in such individuals may not only impair central function but may also serve as a systemic amplifier of aging in peripheral organs [18], including the heart. Second, our model predicts that cancer survivors who undergo cranial irradiation may be at increased risk for accelerated systemic aging and early onset of age-related diseases [90]. This has critical implications for pediatric and adolescent cancer survivors, in whom WBI is used to prevent or treat central nervous system malignancies or leukemia [91]. Clinical observations support this prediction: Hummel et al. reported that childhood cancer survivors who received cranial radiotherapy exhibited long-term cardiac alterations, including reduced cardiac volumes and impaired left ventricular systolic and diastolic function [92]. Importantly, their study also found significantly decreased circulating IGF-1 levels in these patients, supporting the hypothesis that cranial irradiation disrupts hypothalamic-pituitary signaling and contributes to systemic organ aging by suppressing key endocrine factors [92]. In a related study, Brouwer et al. found increased femoral intima-media thickness in childhood cancer survivors who had received localized radiotherapy, suggesting that irradiation can induce systemic vascular changes even outside the primary treatment field [93]. Together, these findings raise the possibility that brain-targeted irradiation accelerates aging-like cardiovascular remodeling through a combination of inflammatory and neuroendocrine mechanisms, underscoring the need for long-term monitoring and targeted interventions in this vulnerable population.

In this context, long-term follow-up studies could be designed to test whether WBI exposure during childhood leads to premature biological aging in adulthood. This could be assessed through emerging biomarkers of senescence, such as elevated levels of circulating SASP components (e.g., IL-6, GDF15, and PAI-1), and through epigenetic aging clocks (e.g., GrimAge, PhenoAge) that reflect accelerated biological age relative to chronological age [94]. Additionally, age-sensitive physiological endpoints—such as decreased cardiorespiratory fitness, early vascular stiffness, impaired diastolic function, or reduced cardiac reserve—could be evaluated as early indicators of cardiovascular aging [95]. Combining molecular biomarkers with longitudinal physiological assessments in cancer survivor cohorts could provide valuable insights into whether early-life brain senescence triggers a systemic pro-geronic state, and whether interventions targeting senescence-related pathways could mitigate these long-term effects.

Despite the strength of the observed transcriptomic overlap, several limitations should be acknowledged. First, while WBI induces widespread brain senescence, it may also trigger secondary changes in behavior, metabolism, or stress responses that could indirectly influence cardiac gene expression. Although the WBI model is targeted to the brain, we cannot fully exclude the possibility of off-target systemic effects, including subtle alterations in hematopoietic or vascular compartments. While shielding minimized direct radiation exposure to peripheral organs, further studies will be required to comprehensively rule out secondary effects outside the CNS. Second, we did not measure circulating SASP factors or perform interventional experiments (e.g., senolytics, parabiosis, and plasma transfer) in this pilot study. Future work should aim to identify the molecular mediators responsible for the brain-to-heart signaling axis, including profiling plasma cytokines and testing whether the elimination of senescent cells in the brain is sufficient to reverse peripheral transcriptomic aging. Another limitation is that bulk RNA sequencing lacks the resolution to attribute transcriptomic changes to specific cardiac cell types. Future studies using single-cell or spatial transcriptomics will be necessary to distinguish cardiomyocyte-specific versus stromal or immune cell contributions to the observed gene expression patterns. Another limitation of the present study is the lack of direct functional assessments of cardiac performance, such as echocardiography or histological fibrosis quantification. As such, conclusions are limited to transcriptomic remodeling and cannot confirm structural or functional impairments at the organ level. An additional important consideration is the timeline of exposure. Our study focused on young animals and examined effects a couple of months after WBI-induced brain senescence. Our analysis was limited to a single post-WBI time point, which precludes conclusions about the persistence, progression, or potential reversibility of the observed transcriptomic effects. It also remains to be determined how prolonged exposure to the altered systemic milieu—driven by persistent brain senescence—may further promote aging in peripheral organs. Over time, additional mechanisms and signaling pathways may be engaged, potentially amplifying or diversifying the systemic aging phenotype. Moreover, although we focused here on the heart due to its known sensitivity to systemic inflammatory and metabolic signals, it is likely that other organs—including skeletal muscle, kidney, and vasculature—are also influenced by brain senescence. Ongoing studies in our group are evaluating multi-organ effects using single-cell and spatial transcriptomics, senescence reporter models, and functional outcome measures.

This pilot study lays the groundwork for deeper investigation into the mechanisms by which brain senescence orchestrates systemic aging. Several key questions remain [96, 97]. Future studies should aim to identify the molecular mediators of brain-to-periphery communication, including detailed profiling of circulating SASP components, neuroendocrine factors, and extracellular vesicles. Interventional strategies—such as brain-targeted senolytic treatments, pharmacological inhibition of the SASP, or genetic ablation of senescent cell populations—will be essential to establish causality and assess whether reversal of brain senescence can mitigate aging phenotypes in peripheral organs. Longitudinal experiments extending beyond the 2-month post-irradiation window used here will help clarify the long-term systemic consequences of persistent brain senescence. In addition, expanding the analysis to other aging-sensitive organs—including skeletal muscle, kidney, liver, and the vasculature—will provide a broader understanding of systemic vulnerability to brain-derived aging cues. Finally, integrating multi-omics approaches (e.g., proteomics, epigenomics, and metabolomics) with transcriptomics will enable a more comprehensive dissection of downstream pathways and cellular targets affected by neurogenic aging signals.

**Fig. 7:**
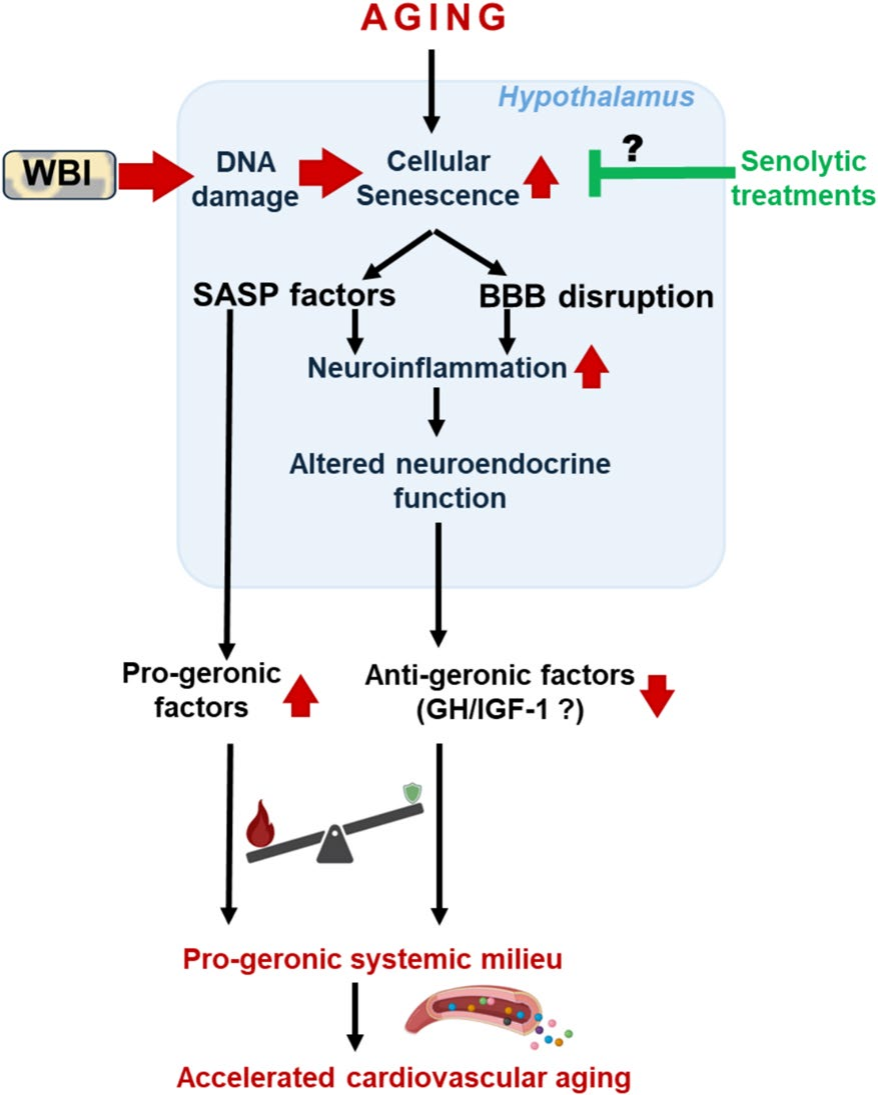
Conceptual model illustrating how brain senescence drives systemic aging. Schematic overview of the proposed mechanism by which localized senescence in the brain—particularly in the hypothalamus—leads to aging-like changes in peripheral organs through cell non-autonomous pathways. With age or following whole-brain irradiation (WBI), microglia and brain endothelial cells acquire a senescence-associated secretory phenotype (SASP), releasing pro-inflammatory cytokines, chemokines, matrix-modifying enzymes, and possibly extracellular vesicles into the brain parenchyma and systemic circulation. Concurrently, brain aging is associated with diminished output of protective neuroendocrine factors such as growth hormone (GH) and insulin-like growth factor-1 (IGF-1), further tipping the balance toward a pro-geronic systemic milieu. These combined effects—an increase in circulating SASP and a decline in anti-aging signals—alter the function and gene expression profiles of distant organs, promoting aging phenotypes in tissues such as the heart, vasculature, and skeletal muscle. The model highlights brain senescence as both a source and amplifier of systemic aging signals, positioning it as a potential upstream target for interventions aimed at extending healthspan

In conclusion, our study demonstrates that localized senescence in the brain is sufficient to drive aging-like transcriptomic remodeling in the heart. These findings identify brain senescence as a central orchestrator of systemic aging and underscore the need to consider the brain not only as a passive target of aging but also as an active source of pro-geronic cues (Fig. 7). Interventions that target senescent brain cells, modulate neuroinflammatory signaling, or neutralize circulating SASP components may represent promising strategies to slow systemic aging and reduce the burden of age-associated diseases.
